# Full-thickness chest wall resection for malignant chest wall tumors and postoperative problems

**DOI:** 10.3389/fonc.2023.1104536

**Published:** 2023-04-21

**Authors:** Kunihiro Asanuma, Masaya Tsujii, Tomohito Hagi, Tomoki Nakamura, Kouji Kita, Akira Shimamoto, Takeshi Kataoka, Motoshi Takao, Akihiro Sudo

**Affiliations:** ^1^ Department of Orthopedic Surgery, School of Medicine, Mie University, Tsu, Japan; ^2^ Department of Thoracic and Cardiovascular Surgery, School of medicine, Mie University, Tsu, Japan

**Keywords:** chest wall, reconstruction, local control, pulmonary function, scoliosis, complication

## Abstract

**Background:**

Chest wall malignant tumor (including primary and metastatic lesions) is rare, representing less than 5% of all thoracic malignancies. Local control of chest wall malignancies requires wide resection with tumor-free margins. These requirements increase the risk of thoracic cavity failure and subsequent pulmonary failure. The restoration strategy for chest wall defects comprises chest wall reconstruction and soft-tissue coverage. Various reconstruction methods have been used, but both evidence and guidelines for chest wall reconstruction remain lacking. The purposes of this study were to collate our institutional experience, evaluate the outcomes of full-thickness chest wall resection and reconstruction for patients with chest wall malignant tumor, and identify problems in current practice for chest wall reconstruction with a focus on local control, complications, pulmonary function and scoliosis.

**Methods:**

Participants comprised 30 patients with full-thickness chest wall malignant tumor who underwent chest wall resection and reconstruction between 1997 and 2021 in Mie University Hospital. All patients underwent chest wall resection of primary, recurrent or metastatic malignant tumors. A retrospective review was conducted for 32 operations.

**Results:**

Recurrence was observed after 5 operations. Total 5-year recurrence-free survival (RFS) rate was 79.3%. Diameter ≥5 cm was significantly associated with poor RFS. The postoperative complication rate was 18.8%. Flail chest was observed with resection of ≥3 ribs in anterior and lateral resections or with sternum resection without polyethylene methylmethacrylate reconstruction. Postoperative EFV1.0% did not show any significant decrease. Postoperative %VC decreased significantly with resection of ≥4 ribs or an area of >70 cm2. Postoperative scoliosis was observed in 8 of 28 patients. Posterior resection was associated with a high prevalence of scoliosis (88.9%).

**Conclusion:**

With chest wall reconstruction, risks of pulmonary impairment, flail chest and scoliosis were significantly increased. New strategies including indications for rigid reconstruction are needed to improve the outcomes of chest wall reconstruction.

## Introduction

Malignant tumor of the chest wall, including primary and metastatic lesions, is rare and the incidence is below 5% of all thoracic malignancies (1). For primary sarcomas, less than 20% arise in the chest wall (2). Surgical treatment for chest wall malignant tumor requires special knowledge and techniques because of the anatomical and structural particularities (3). Local control of chest wall malignancy needs wide resection with tumor-free margins (4). Wide resection of the chest wall, however, risks thoracic cavity failure and subsequent pulmonary failure.

The restoration strategy for chest wall defects involves chest wall reconstruction and soft-tissue coverage. In preparing for chest wall reconstruction, the resection area, stability of the chest wall, organ protection, respiratory function, organ herniation, scapular trapping, reconstruction materials and other issues need to be considered. Various technological advancements have provided materials for use in multiple modes of reconstructive surgery. For non-rigid reconstruction, synthetic meshes like polypropylene (5), polyester (6), polytetrafluoroethylene (PTFE) (7), vicryl (8) and biological tissues such as fascia lata (9) are usually used. For rigid reconstruction, sandwich methods using polyethylene methylmethacrylate (PMMA) and polypropylene mesh (10), titanium plates (11), allografts (12), and various three-dimensionally printed implants (13) are used. These different options offer significant benefits to the patient in terms of preserving quality of life. However, the indications for non-rigid or rigid reconstruction have not been clearly defined and neither accumulated evidence nor guidelines have been presented to address this problem (14). In addition, each reconstruction method involves issues of insufficient restoration and potential adverse effects. In our institute, polypropylene, PTFE or flaps have been used for non-rigid reconstructions, and the sandwich method for rigid reconstruction. The purposes of this study were to collate our institutional experience, evaluate the outcomes of full-thickness chest wall resection and reconstruction for patients with chest wall malignant tumor, and suggest considerations for current chest wall reconstructions with a focus on local control, complications, pulmonary function and scoliosis.

## Methods

A total of 30 patients with chest wall malignant tumor who underwent chest wall resection and reconstruction between 1997 and 2021 at Mie University Hospital were enrolled in this study. All patients had undergone full-thickness chest wall resection of primary, recurrent or metastatic malignant tumors. Histopathological diagnoses were verified by independent pathologists. A retrospective review of all patients was performed using data collected from hospital records and follow-up information. This study was approved by the Ethics Committee of the Mie University Graduate School of Medicine (approval number: H2020-224). All procedures performed in studies involving human participants were undertaken in accordance with the ethical standards of the Ethics Committee of Mie University and with the 1975 Declaration of Helsinki.

For the purposes of defining locations, the anterior area was defined as between the sternum and the anterior axillary line, the lateral area was defined as between the anterior and posterior axillary lines, and the posterior area was defined as between the spine and the posterior axillary line.

Reconstruction was categorized as involving no prosthesis, nonrigid prosthesis or rigid prosthesis. Cases with no prosthesis were closed using residual tissues or transferred flap. Nonrigid prosthetic reconstruction used Marlex mesh (Bard, Cranston, RI) or BARD mesh. Excess mesh was trimmed and then 1-0 nylon sutures were tightened between bone or soft tissue and the mesh as much as possible. Rigid prosthetic reconstruction was performed using a modified sandwich method with Marlex mesh and PMMA (Simplex P; Stryker Howmedica Osteonics, Mahwah, NJ). PMMA was molded smaller than the chest defect by hand on a back table and applied within a double-layer or quadri-layer mesh. Mesh around the PMMA was sutured to fix the prosthetic in location using 3-0 proline or nylon to allow movement of PMMA inside the mesh. Excess mesh was trimmed and 1-0 nylon sutures were tightened between the bone or soft tissue and mesh. The choice of reconstruction method was decided based on the location, defect size and number of ribs resected. Most resections involving 3 or more ribs were reconstructed with mesh. Sandwich reconstruction with mesh and PMMA was used for cases of sternum resection, lateral 7-rib resections or re-resection of ribs due to recurrence.

Preoperative and post operative pulmonary function testing was performed in 16 operations. Pulmonary functions evaluated included percentage predicted forced expiratory volume in 1 s (FEV1.0%, FEV1/FVC) and % vital capacity (%VC, VC/predicted VC). %VC ratio was calculated by following formula. %VC ratio = (postoperative %VC)/(preoperative %VC). During follow-up evaluations, scoliosis was diagnosed as a thoracic Cobb angle ≥10° on X-ray or CT. For this study, maximum Cobb angle was noted for 1 and 5 years postoperatively and as of the most recent follow-up.

### Statistical analysis

Statistical analyses were performed to compare various parameters using the Mann-Whitney test or Kruskal-Wallis test for quantitative data and Fisher’s exact test for qualitative data. Local recurrence-free survival (RFS) was defined as the time from operation to the date of clinically documented local recurrence. Kaplan-Meier survival plots and log-rank tests were used to assess differences in time to local recurrence. Values of p<0.05 were considered statistically significant. The EZR software program was used for all statistical analyses (15).

## Results

### Patient and tumor characteristics

Mean age of the 30 patients (17 males, 13 females) was 53.8 years (range, 1–78 years). Mean tumor size was 8.0 cm (range, 2.5–23 cm). Histopathological diagnoses are shown in **Table 1**. One patient underwent 3 resections, for the primary tumor and two recurrences. We analyzed a total of 32 operations, comprising 17 operations for primary tumors, 3 operations for recurrent tumors, 8 operations for metastatic tumors and 4 operations for tumors that had been resected inadequately in a previous hospital (**Table 2**). All patients received wide resection. Mean duration of follow-up was 133 months (range, 1.2–240 months).

**Table 1:** Histopathological diagnosis.

	Histology	30
Primary tumor		
	Chondrosarcoma	5
	Leiomyosarcoma	5
	Ewing sarcoma	3
	Myxofibrosarcoma	2
	Solitary fibrous tumor	2
	Rhabdomyosarcoma	1
	Malignant peripheral nerve sheath tumor	1
	Osteosarcoma	1
	UPS	1
	Spindle cell sarcoma	1
Metastatic tumor		
	Hepatocellular carcinoma	2
	Renal cell carcinoma	2
	Meningioma	1
	Osteosarcoma	1
	Leiomyosarcoma	1
	Synovial sarcoma	1

Histopathological diagnosis was shown.

**Table 2:** Surgical data 1.

	Histology	30
Primary		17
Recurrence		3
Metastasis		8
After resection in previous hospital		4
Resected bone	1 Rib	8
	2 Ribs	8
	3 Ribs	7
	4 Ribs	3
	7 Ribs	2
	Sternum	4
Location	Ante	6
	Late	16
	Post	10

Surgical information was shown. Ante: anterior resection, Late: lateral resection, Post: posterior resection.

### Surgical data

A total of 32 operations were reviewed. Rib resection including pleura was performed in 28 operations and sternum resection was performed in 4 operations. The number of resected ribs is shown in **Table 2**. Resected area was anterior in 6 operations, lateral in 16 and posterior in 10. Simple wound closure was performed in 17 operations. Reconstruction by mesh was used in 5 operations, mesh and flap in 7 operations and mesh, flap and PMMA in 3 operations. Flaps used were latissimus dorsi (LD) flap in 9 operations and pectoralis major flap in 1. Mean operation time was 4.9 h (range, 1.5–11 h) and mean volume of blood loss was 432 ml (range, 43–3170 ml). Adjuvant radiotherapy was performed after 6 resections (**Table 3**).

**Table 3:** Surgical data 2.

Surgical methods	Simple closure	17
	Mesh	5
	Mesh+flap	7
	Mesh+flap+PMMA	3
Flap	latissimus dorsi	9
	pectoralis major	1
Operation time		1.5-11 h (mean: 4.8 h)
Bleeding		43-3170 ml (mean: 432 ml)
Adjuvant radiotherapy		6

Surgical data about reconstruction, operation time, bleeding and radiotherapy was shown.

### Five-year RFS

**Figure 1:**
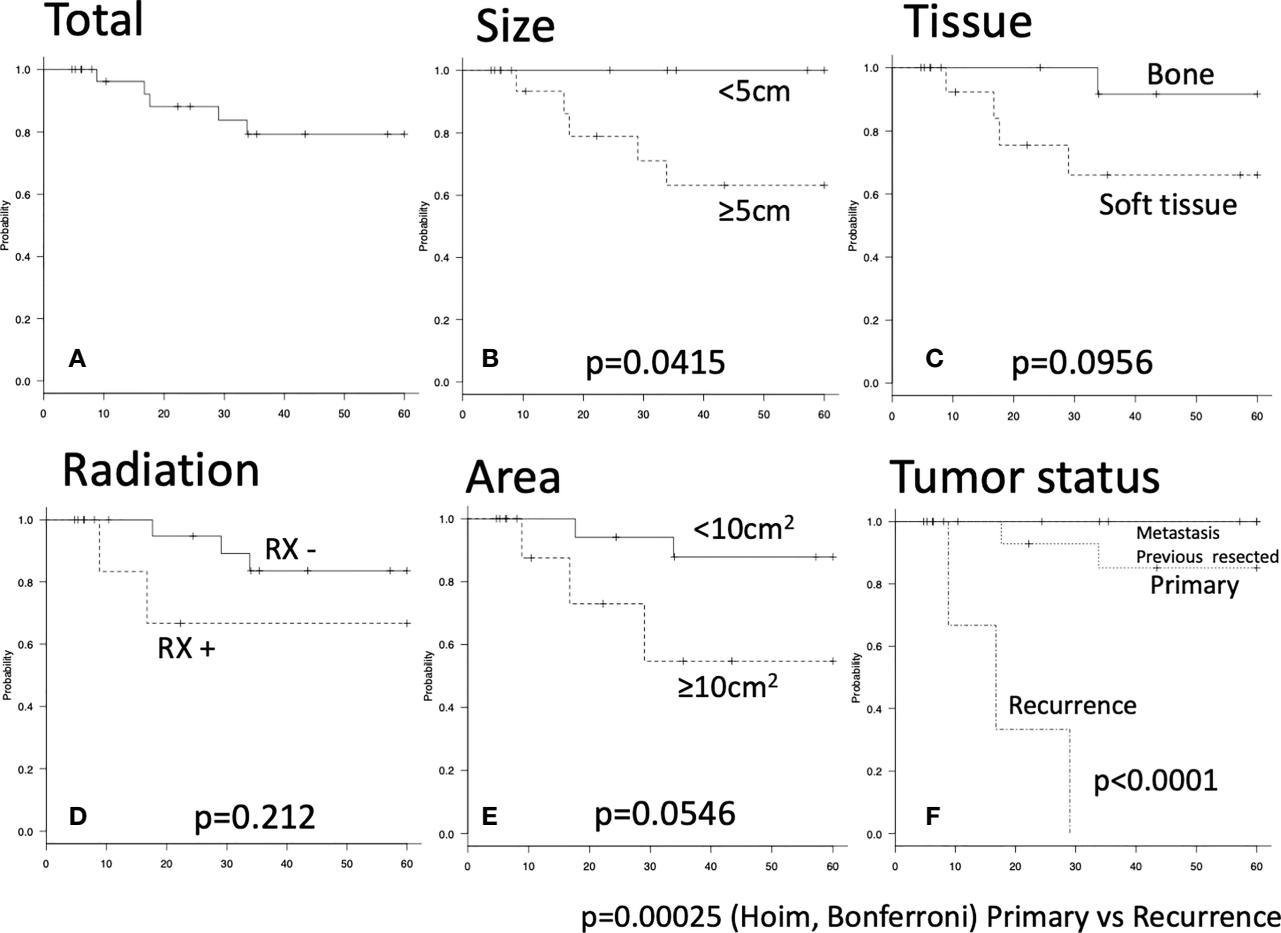
Five-year recurrence-free survival were shown by Kaplan-Meier carve. The log-rank p value of less than 0.05 was considered as statistically significant. **(A)** Total five-year recurrence-free survival. **(B–E)** There were no significant differences. **(F)** Significant difference was seen between primary and recurrence group by Holm and Bonferroni test (p<0.001).

Recurrence within 5 years was observed for 5 operations. Total 5-year RFS rate was 79.3% (**Figure 1A**). Tumor diameter ≥5 cm was significantly associated with poor RFS (**Figure 1B**). 5-year RFS rate for tumor diameter <5 cm: 100%; 5-year RFS rate for tumor diameter ≥5 cm: 63.2%; p=0.0415). A tendency toward higher risk of recurrence was seen for soft-tissue tumor (**Figure 1C**; 5-year RFS rate for bone tumor: 91.7%; 5-year RFS rate for soft-tissue tumor: 66.1%; p=0.0956), adjuvant radiation (**Figure 1D**; 5-year RFS rate with radiation: 83.6%; 5-year RFS rate without radiation: 66.7%; p=0.212), and resected area ≥10 cm2 (**Figure 1E**; 5-year RFS rate with resected area <10 cm2: 87.8%; 5-year RFS rate with resected area ≥10 cm2: 54.7%; p=0.0546). Recurrent tumors also showed an elevated risk of further recurrence (**Figure 1F**).

### Pulmonary function

**Figure 2:**
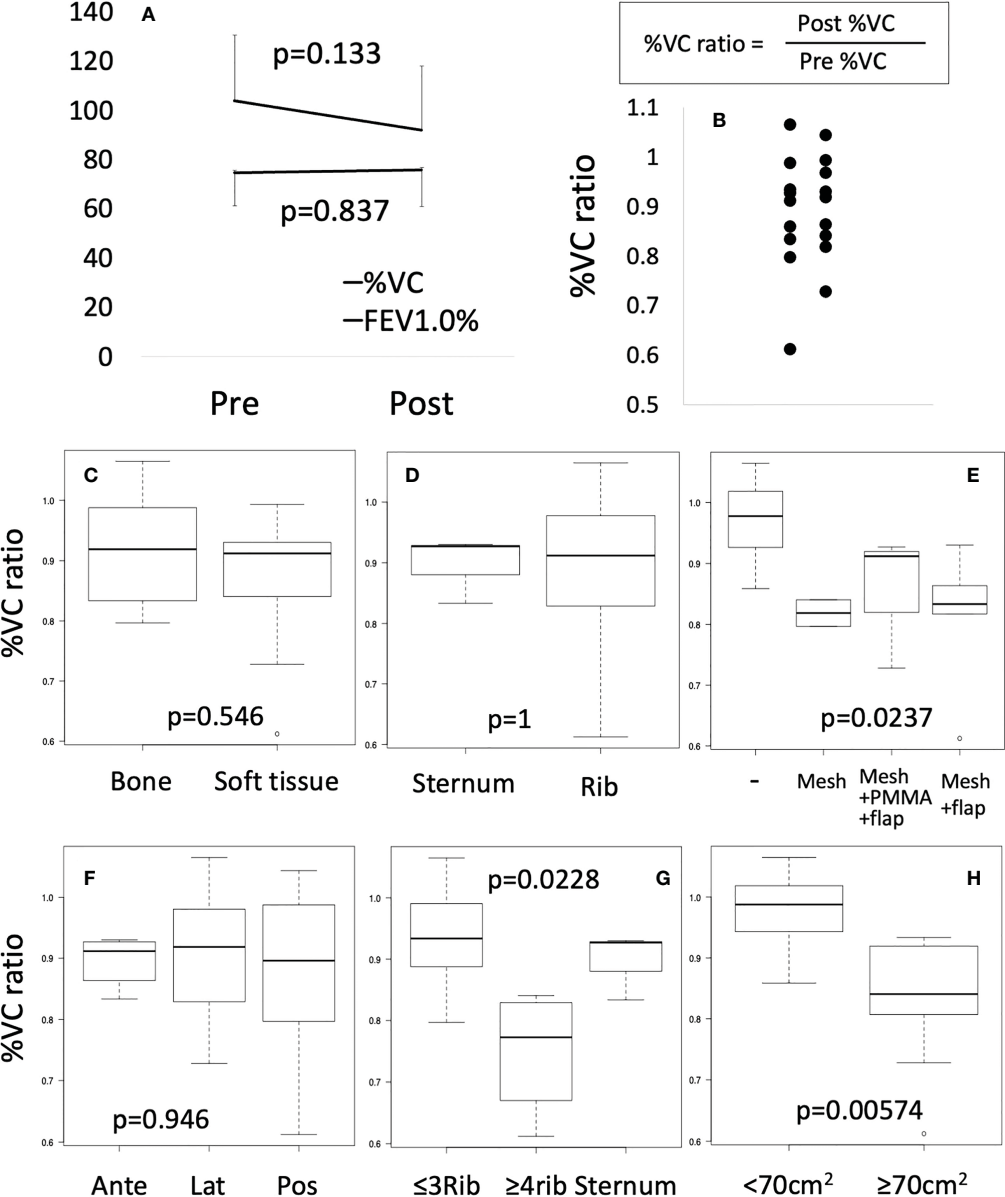
Pulmonary function test. **(A)** Preoperative (Pre) or postoperative (Post) %VC and FEV1.0% were compared by Mann-Whitney test. **(B)** %VC ratio was shown by scatter plot. **(C–H)** Box plots were shown. Mann-Whitney test or Kruscal-Wallis test were performed.

Comparing pulmonary function between pre- and postoperatively, mean FEV1.0% was not significantly different. Since mean %VC tended to decrease (p=0.113) (**Figure 2A**), only %VC values were used for further analysis. %VC ratio showed a distribution from 0.61 to 1.04 (**Figure 2B**). %VC ratio between bone and soft tissue (**Figure 2C**), sternum and rib (**Figure 2D**) did not show significant difference. Based on the method of reconstruction, %VC ratio seemed to be decreased for mesh and mesh plus flap reconstructions, although the difference was not significant by Holm or Bonferroni test (**Figure 2E**). Tumor location did not affect %VC ratio (**Figure 2F**). The %VC ratio was significantly decreased with resection of ≥4 ribs (p=0.0228) and resection of ≥70 cm2 (p=0.00574) (**Figures 2G, H**).

### Complications

Complications were observed following 6 of the 32 operations (18.8%). Infection was seen in 1 case that underwent resection of 7 ribs for a 10-cm leiomyosarcoma in the posterior area. Reconstruction was performed using Marlex mesh covered by transferred LD muscle. Infection developed in that case 1 week postoperatively and the surgical wound was opened over the LD layer. With washing, antibiotics and use of a negative-pressure wound therapy, the infection resolved and the wound was completely closed within 2 months. Cardiac effusion was seen as a complication in 1 case after sternum resection due to metastasis of synovial sarcoma. The chest wall was reconstructed using Marlex mesh and PMMA, then covered by transferred LD muscle. Two months after the operation, asymptomatic cardiac effusion was observed on CT. Cytology of the fluid showed no malignancy and no recurrence of cardiac effusion was observed. Hematoma was seen 2 cases. In one case, a 12-cm antero-lateral leiomyosarcoma was resected, including 3 ribs. The patient complained of dyspnea 3 days after the operation. Hematoma was then detected in the thoracic cavity on CT. Reoperation was performed to check for bleeding sites, but no obvious bleeding was observed. Postoperatively, no further bleeding was observed. In the other case, a 9-cm myxofibrosarcoma was resected along with 4 ribs. Reconstruction was performed using mesh and LD flap. On postoperative day 20, the patient complained of dyspnea and CT showed fluid in the thoracic cavity. A thoracic drainage tube was inserted for 5 days and the patient recovered. Respiratory failure occurred as a complication in 1 case. After resection of the sternum for a metastatic brain tumor, the chest wall was reconstructed using Marlex mesh and covered by transferred LD muscle. Respiratory failure developed after extubation and noninvasive positive-pressure ventilation was initiated. After 12 h, respiratory failure had fully recovered. Partial flap necrosis was seen as a complication in 1 case. After a 23-cm chondrosarcoma was resected with 4 ribs, the chest wall was reconstructed using BARD mesh and covered by transferred LD muscle. A small area of necrotic flap was observed and debridement and wound closure were performed 6 days after resection. Previous research has reported postoperative complication rates of 22–33.2% (16–18), comparable to our complication rate of 18.8%.

### Flail chest

Chest wall motion was tested by forced deep breathing. Chest wall depression was checked for by inspection or palpation. Slight movement of the chest wall was suspected to represent flail chest. 20 operations were evaluated for flail chest from the medical records or medical examinations and 5 cases of flail chest were detected. All 5 cases of flail chest occurred with mesh reconstructions, and no cases of flail chest were not detected in reconstructions using PMMA (**Table 4A**, p=0.00774). In the 13 operations without PMMA reconstruction, flail chest was observed in cases with resection of ≥3 ribs or the sternum (**Table 4B**, p=0.0256). In terms of the region of resection, most cases of flail chest involved anterior or lateral resections (**Table 4C**, p=0.0124).

**Table 4:** Flail chest.

A	N=20	Flail -	Flail +	
	–	9	0	P=0.00774
	Mesh	3	5	
	Mesh+PMMA	3	0	
B	N=16	Flail -	Flail +	
	≤2Ribs	7	0	P=0.0256
	≥3Ribs	4	3	
	Sternum	0	2	
C	N=16	Flail -	Flail +	
	Ante	0	3	P=0.0124
	Late	6	2	
	Post	5	0	

Surgical data of flail chest about reconstruction, resected bone and resected area was shown.

### Postoperative scoliosis

**Figure 3:**
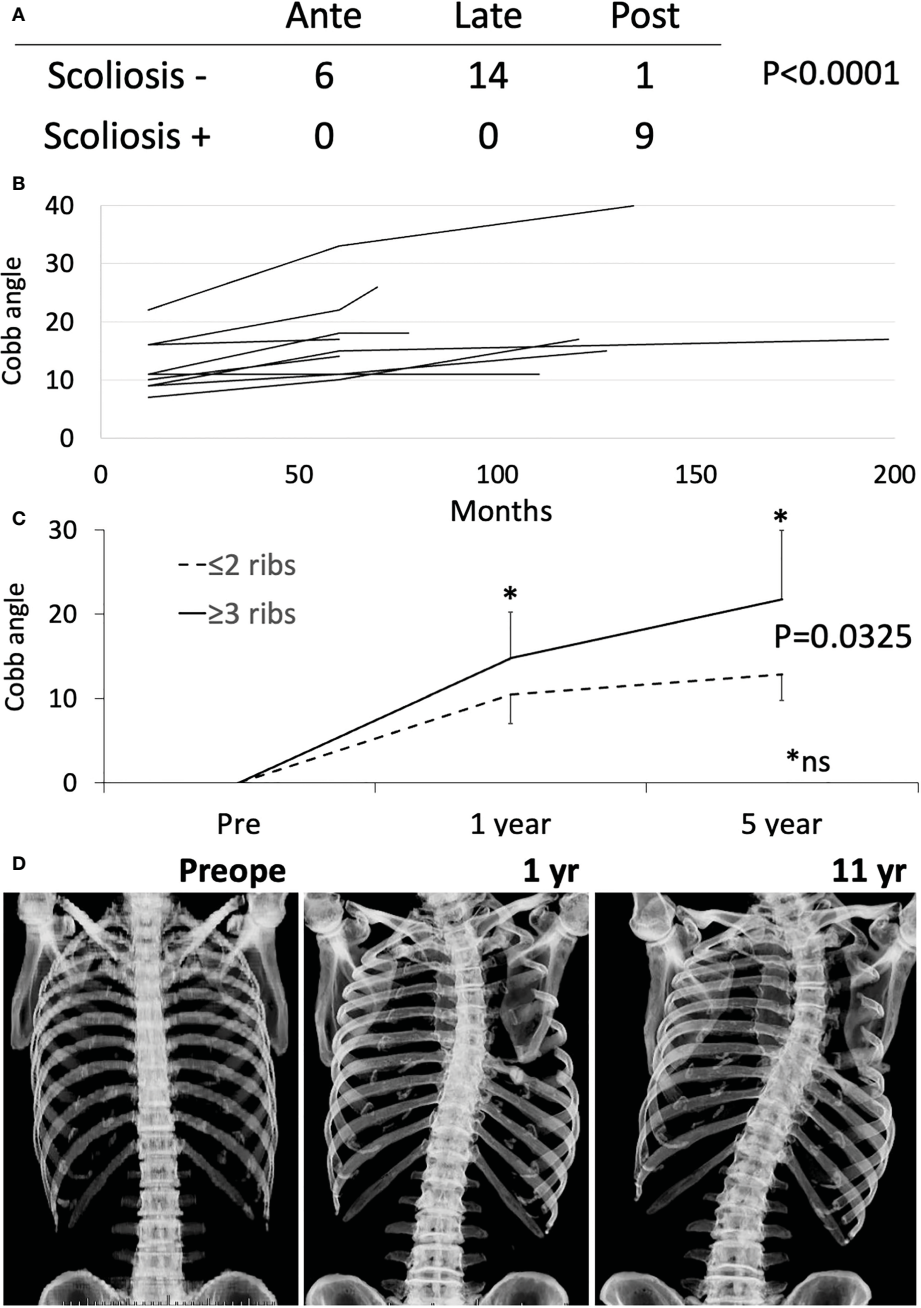
Scoliosis analysis. **(A)** Fisher exact test was performed between anterior (Ante), lateral (Late) and posterior (Post) resection with or without scoliosis. **(B)** Cobb angle at 1 year, 5 years, and at last follow-up was shown by the line chart. **(C)** The time-dependent changes of cobb angle were shown between groups with resection of ≤2 ribs and ≥3 ribs. The time-dependent analysis showed significant difference by repeated-ANOVA (p=0.0325). * Mann-Whitney test at 1 year and 5 year time point was not significant (ns). **(D)** Chest Xray of a representative case is shown.

Scoliosis is a potential long-term complication. Scoliosis was diagnosed by comparing X-ray or CT images before and after the operation, and was defined as a Cobb angle ≥10°. Cobb angle was measured at 1 year, 5 years, and at last follow-up. Postoperative scoliosis was diagnosed in 9 of the 28 patients for whom imaging data were available, and posterior resection was associated with a higher incidence of scoliosis (**Figure 3A**, p<0.0001). In patients with posterior resection, scoliosis was observed in 90% (9/10) of patients with resection of 1, 2, 2, 2, 2, 3, 3, 4 and 7 ribs (**Figure 3B**). One patient was excluded because of vertebral fixation. At 1 year postoperatively, Cobb angle ≥10° was observed in 5 patients (with resection of 2, 2, 3, 4 and 7 ribs). At 5 years, Cobb angle ≥20° was seen in patients who underwent resection of 4 and 7 ribs. Mesh reconstruction of posterior resection was performed in 4 cases and seemed to be of no help in preventing scoliosis. Cobb angle at 1 and 5 years timepoint was compared between groups with resection of ≤2 ribs and ≥3 ribs postoperatively, showing no significant difference (**Figure 3C**, Mann-Whitney test). ≥3 ribs resection group showed significantly worse cobb angle than ≤2 ribs resection group by the time-dependent analysis (**Figure 3C**, repeated-ANOVA, p=0.0325). A representative case is shown in **Figure 3D**. A 66-year-old man underwent chest wall resection due to a 10-cm leiomyosarcoma. Seven ribs were resected posteriorly and the defect was reconstructed using Marlex mesh and transferred LD muscle. Scoliosis was observed 1 year postoperatively and gradually progressed (**Figure 3D**).

## Discussion

Surgical resection for chest wall malignant tumors is a most reliable treatment. As our study included treatment for chest wall metastases, only local control was evaluated. According to previous researchers, the local recurrence rate was 35% in 3 years among patients with sarcoma (19), 25% at 5 years among patients with sarcoma or cancer (1), 11% at 5 years among patients with sarcoma (5-year RFS, 88.5%) (20) and 59% at 10 years with sarcoma or cancer (4). Several studies have suggested risk factors for local recurrence. High-grade sarcoma was significantly associated with recurrence (21). Relapse correlated directly with R1 disease (4). The recurrence rate of R2 resection reportedly approaches 100% (22). In our study, the 5-year RFS rate was 79.3% and the local recurrence rate was 15.6% (5/32). Risk factor analysis showed tumor size ≥5 cm was significantly associated with poorer RFS. This study successfully demonstrated that our local control rate was not inferior to previous studies and tumors ≥5 cm had a greater potential for recurrence.

The objective of chest wall reconstruction after chest wall resection is to preserve normal chest wall mechanics and pulmonary function while avoiding flail chest. To approximate the resected chest wall to the preoperative state, chest wall reconstruction has been performed using rigid or non-rigid methods, but the topic remains controversial. Indications for different methods have not been clearly delineated and no accumulated evidence or guidelines have been provided for this problem. Many authors have reported good outcomes from non-rigid reconstruction with suture stabilization (23), flap, fascia lata (9) and mesh (7, 24). According to the authors of a report on rigid reconstruction, anterior rib or sternum defects, or a minimum of 3 anterolateral ribs were considered an indication for rigid reconstruction (10, 25). In an expert consensus, this was raised as an agenda in consensus 5. In the survey of the report, 71.4% of surgeons indicated they always used rigid reconstruction for chest wall defects >5 cm (14). PMMA plus mesh has been used clinically since the 1970s (26). This method has been a popular choice for chest wall reconstruction for many decades. With advances in technology, various materials have been developed for rigid reconstruction. Titanium plates have gained popularity and often require combined use of other reconstructive methods (11, 25, 27). Various 3-dimensionally printed implants are now being developed (13, 28). In an expert consensus survey, 47.6% of surgeons used titanium plates and 15.4% used PMMA plus mesh or other implants (14).

In terms of the choice of implant, long-term stability and implant survival are important problems. In papers on chest wall reconstruction using titanium plates, Berthet et al. reported 2 cases with plate fracture (10.5%) and De Palma et al. reported 2 cases of plate fracture and 1 case of pin dislodgment (11.1%) (11, 27). Furthermore, Berthet et al. reported 20 broken plates and 4 displaced plates (44%), and the survival rate of plates was around 50% within 2 years (29). Such data suggest that titanium plates do not offer good long-term stability for chest wall reconstruction. Basically, titanium is a bioinert material that does not form a biological connection between the plate and soft tissue or bone (30). The strength of plate fixation by screws or clips thus depends on bone strength. In particular, fixation strength would be low for patients with osteoporosis, such as elderly women. The data on pedicle screw loosening into osteoporotic vertebral bodies support this contention (31, 32). Failure of the bone and screw interface, mostly bone, leads to plate displacement. In addition, in terms of plate breakage, studies examining implant failure with nonunion of bone are warranted. Bridging *via* a metal device without bone union provides foci of mechanical stress, leading to concentration of the kinetic load and eventual device breakage due to metal fatigue (33). Given this, for plate reconstruction without biological connection to bone, long-term stability of the plate fixation cannot be expected.

Our opinion regarding the ideal material for chest wall reconstruction is that its properties should include an adequate shape for the defect, sufficient rigidity to prevent flail chest and protect against intrathoracic migration of organs, durability, and biologically inertness for organs but biologically compatibility with surrounding connective tissues. For long-term stability, biological integration of the implant is a significant issue. We have previously reported soft-tissue integration with polypropylene mesh from an analysis using resected tissues in which soft tissue sarcoma recurred after reconstruction using BARD mesh (30). The sandwich method we prefer has two advantages: rigidity from the PMMA; and biological integrity from the polypropylene mesh. Using the sandwich method, polypropylene mesh around PMMA is integrated with soft tissues and connects to the chest wall biologically. This biological integration may be one reason for the low incidence of implant dislocation with the sandwich method (1.3%, 6/457) (34).

In addition, maximal expiratory and inspiratory pressure from respiratory muscles was 180 cmH_2_O at most for males (35). To prevent flail chest, fixation strength to endure a pressure of around 180 cmH_2_O is thought to be sufficient. Various modified sandwich methods have been reported and we used our own version. PMMA was molded smaller than the chest defect and mesh around the PMMA was sutured to fix the location using 3-0 proline or nylon. The excess mesh was cut off and 1-0 nylon sutures were placed circumferentially between bone or soft tissue and the mesh, then tightened as much as possible. Flexibility between bone and PMMA is expected to disperse the concentration of mechanical stress.

As next-generation materials for chest wall reconstruction, tailored 3-dimensional printed implants have been attracting attention. To date, titanium and its alloys are major materials in various reconstructions, including of the chest wall (36). Titanium and its alloys are very unique materials in which surface modifications change the ability for tissue connection. Titanium and titanium alloys are thought to be bioinert for bone connection and smooth surfaces cannot connect to bone or soft tissue. On the other hand, a rough surface may allow connection (30, 37, 38). Surface modification at the site of proposed tissue connection to the chest wall edge may thus allow biological integration and improved durability.

Respiratory function after chest wall resection has been assessed in several studies. Marulli et al. performed reconstructions using allograft sternochondral replacement after sternectomy. Respiratory functions were examined in the first 7 patients and did not show deterioration of respiratory function (12). Daigeler et al. reported chest wall reconstruction with or without prolene mesh or flap in 92 patients, with pulmonary function tested for 27 patients. The %VC was reduced to a mean of 94.9% of the predicted value (range, 42.8–130.9%), and %FEV1 was decreased to 81.8% (range, 46.3–120.1%) compared to expected values (39). Lardinois et al. reported chest wall reconstruction using PMMA and mesh in 26 patients. %FEV1 did not show any significant difference between the periods pre- and postoperatively (40). Leuzzi et al. reported chest wall reconstruction with or without vicryl mesh or expanded polytetrafluoroethylene in 175 patients. Both %FEV1 and %FVC tended to be slightly reduced from 87.1 ± 18.9% preoperatively to 82,3 ± 23.0% postoperatively and from 94.1 ± 19.3% preoperatively to 82.0 ± 21.6% postoperatively. Reduction of %FEV1 in a group with no prosthesis stabilization (17.5 ± 16.2%) tended to be worse than in a group with prosthesis reconstruction (4.1 ± 15.9%; p=ns) (8). In this study, %VC and %FEV1 did not differ significantly between those pre- and postoperatively. Focusing on a slight postoperative reduction in %VC, subgroup analysis showed postoperative %VC was significantly lower with resection of >4 ribs or an area >70 cm^2^ compared to preoperatively. Leuzzi et al. showed that %FEV1 was significantly reduced with anterolateral resection (p=0.026). Overall, reconstruction after chest wall resection did not result in a significant deterioration in pulmonary function. However, high-risk groups for %VC reduction such as patients with resection of >4 ribs or an area >70 cm^2^ and anterolateral resection may need more careful planning of the operation and treatment. Rigid reconstruction may help preserve %VC for such high-risk cases.

Scoliosis after chest wall resection should be considered and various risk factors for scoliosis were reported (22, 41–43). In terms of the region of resection, the incidence of scoliosis was higher with posterior resection than with lateral or anterior resection (22, 41, 43). In terms of resection level and depth, procedures superior to the sixth rib or with pleural resection have shown an elevated risk of scoliosis (42). The resection of ≥3 ribs in the posterior region correlated with scoliosis development and the number of ribs resected was related to the degree of curvature (41, 43). The direction of convexity is toward the side of resection more often than away from the side of resection. All patients in a study by Scalabre et al. (43) and 8 of 11 patients in a study by Glotzbecker et al. (42) showed convexity toward the side of resection. Most authors have reported higher risks of scoliosis developing in operations for children and adolescents (22, 41–43). However, no reports have clarified the risk of scoliosis in adults. In our study, scoliosis developed in 88.9% (8/9) of posterior resections and ages at resection were 1, 17, 26, 50, 57, 63, 66, and 69 years in those 8 cases. All curves were convex toward the side of resection. The number of resected ribs was related to severity of Cobb angle at 1 and 5 years after resection. Scoliosis is more likely after wide posterior resection not only in children, but also in adults. Advice on scoliosis prevention has not identified preferable reconstruction methods. In children, many surgeons avoid rigid reconstruction that may hinder correct growth of the spine, because of adaptation problem between skeletal growth and implanted materials. Puviani et al. recommended using fascia lata to allow natural adaptation to the still growing chest of young patients (9). In our series, scoliosis was observed in 88.9% of posterior rib resections, even with resection of only 1 rib, and Cobb angle worsened with a greater number of resected ribs. Convexity toward the side of resection means a loss of longitudinal musculoskeletal support. The optimal procedure to address this has not been elucidated and rigid reconstruction has been proposed in an expert consensus report without due evidence (14). Longitudinal rigid fixation may prevent dilation between the upper and lower rib spaces and may inhibit or alleviate scoliosis. Traditionally, reconstruction of posterior defects is not necessary to prevent flail chest or protect the intrathoracic organs, except with defects over 10 cm and scapular trapping. However, rigid reconstruction may be helpful for posterior defects to prevent scoliosis. The efficacies of different methods for inhibiting scoliosis have not been reported and further clinical studies are needed.

## Conclusion

Total 5-year RFS rate was 79.3% and the complication rate was 20%. Tumor diameter ≥5 cm and recurrent tumor showed poorer RFS. Both %VC and %FEV1 were preserved postoperatively, but %VC was significantly decreased with resection of ≥4 ribs or defect size ≥70 cm^2^. Posterior resection was associated with a higher incidence of scoliosis developing, including in adults. Further studies are needed to clarify whether rigid reconstruction may help prevent reductions in %VC or development of scoliosis.

### Limitation

Our study had the following limitations. This study was a retrospective series of cases from a single institution. The number of patients was small. Statistical analysis could not be performed for each pathological subtype due to the small numbers of cases. However, we reported our experience with the treatment of malignant tumors of the chest wall and postoperative problems. We believe that this study provides worthwhile additional data for chest wall resection and reconstruction surgery.

## Data availability statement

The raw data supporting the conclusions of this article will be made available by the authors, without undue reservation.

## Ethics statement

The studies involving human participants were reviewed and approved by Ethics Committee of the Mie University Graduate School of Medicine (approval number: H2020-224). Written informed consent to participate in this study was provided by the participants’ legal guardian/next of kin.

## Author contributions

KA wrote the main manuscript text and prepared all figures and tables. MTs, TH, KK, and TK extracted the information from the databases. TN and ASh analyzed the data. ASu and MTa supervised the entire study. All authors contributed to the article and approved the submitted version.
